# Supplementary material for: Electron fourier ptychography for phase reconstruction
Source: Sci Rep. 2025 Oct 30;15:37955. doi: 10.1038/s41598-025-21638-7 (PMC12575875; doi:10.1038/s41598-025-21638-7)
Supplement: Supplementary file 1 — Supplementary Material 1 [file 41598_2025_21638_MOESM1_ESM.docx]

Supplementary Material for

**Electron Fourier ptychography for phase reconstruction**

Jingjing Zhao *et al.*

Corresponding author: Jingjing Zhao, [jingjing.zhao@rfi.ac.uk](mailto:jingjing.zhao@rfi.ac.uk)

**This file includes:**

Supplementary Text S1 to S4

Figs. S1 to S10

Tables S1 to S2

References (1 to 9)

Supplementary Text

Text S1. Effective wave transfer function

The effective wave transfer function $w^{'}\left( \boldsymbol{k},\boldsymbol{k}_{\boldsymbol{\tau}} \right)$ under tilted illumination is described by^1,2^:

$w^{'}\left( \boldsymbol{k},\boldsymbol{k}_{\boldsymbol{\tau}} \right)= E_{t}^{'}\left( \boldsymbol{k},\boldsymbol{k}_{\boldsymbol{\tau}} \right)E_{s}^{'}\left( \boldsymbol{k},\boldsymbol{k}_{\boldsymbol{\tau}} \right)exp(-i\chi^{'}\left( \boldsymbol{k},\boldsymbol{k}_{\boldsymbol{\tau}} \right))$ (1)

where $\chi^{'}\left( \boldsymbol{k},\boldsymbol{k}_{\boldsymbol{\tau}} \right)$ is the effective aberration function, and $E_{t}^{'}\left( \boldsymbol{k},\boldsymbol{k}_{\boldsymbol{\tau}} \right)$ and $E_{s}^{'}\left( \boldsymbol{k},\boldsymbol{k}_{\boldsymbol{\tau}} \right)$ are the partial temporal and spatial coherence envelope functions. In Eq.(1), $\boldsymbol{k}$ is a two-dimensional vector in reciprocal space, and $\boldsymbol{k}_{\boldsymbol{\tau}}$ is the two-dimensional wave vector of the tilted incident beam.

These three functions are given by:

$\chi^{'}\left( \boldsymbol{k},\boldsymbol{k}_{\boldsymbol{\tau}} \right) =\chi\left( \boldsymbol{k}+\boldsymbol{k}_{\boldsymbol{\tau}} \right)-\chi(\boldsymbol{k}_{\boldsymbol{\tau}})$ (2)

$E_{t}^{'}\left( \boldsymbol{k},\boldsymbol{k}_{\boldsymbol{\tau}} \right)=exp\left\{ -{(\frac{\pi\Delta\lambda}{2})}^{2}\left[ {(\boldsymbol{k}+\boldsymbol{k}_{\boldsymbol{\tau}})}^{2}-{\boldsymbol{k}_{\boldsymbol{\tau}}}^{2} \right]^{2} \right\}$ (3)

$E_{s}^{'}\left( \boldsymbol{k},\boldsymbol{k}_{\boldsymbol{\tau}} \right)=exp\left\{ -{(\frac{\theta}{2\lambda})}^{2}\left| \boldsymbol{\nabla}\chi^{'}\left( \boldsymbol{k},\boldsymbol{k}_{\boldsymbol{\tau}} \right) \right|^{2} \right\}$ (4)

where $\Delta$is the e^-1^ half-width value of the focal spread distribution, $\theta$ is the illumination semiangle and $\lambda$ is the electron wavelength. Details of the effective aberration coefficients have been previously described^1–3^. Here we define an effective image shift $A_{0}^{'}$ and effective defocus $C_{1}^{'}$ as used in the main text as:

$A_{0}^{'}=A_{0}+ A_{1}\tau^{*}+C_{1}\tau+A_{2}\tau^{*2}+\frac{1}{3}B_{2}^{*}\tau^{2}+\frac{2}{3}B_{2}\tau^{*}\tau+C_{3}\tau^{*}\tau^{2}$ (5)

$C_{1}^{'}=C_{1}+\mathrm{Re}(\frac{4}{3}B_{2}\tau^{*})+2C_{3}\tau^{*}\tau$ (6)

where $A_{0}$, $A_{1}$, $C_{1}$, $A_{2}$, $B_{2}$, and $C_{3}$ are axial aberration coefficients corresponding to image shift, two-fold astigmatism, defocus, three-fold astigmatism, axial coma, and spherical aberration, respectively.

This notation of the aberration coefficients follows the convention given by Saxton^3^ and we write the beam tilt $\tau$ in complex form as $\tau=t_{x}+it_{y}$.

Text S2. Iterative reconstruction

The update steps for one iteration of the modified Ptychographic Iterative Engine (PIE)^4,5^ algorithm used in this work are similar to those in conventional PIE-base ptychographic reconstruction. The key difference is that, in Fourier ptychography, the amplitude update of the image wave occurs in real space, while the exit wave update takes place in Fourier space. In contrast, conventional ptychographic reconstruction updates the image wave in Fourier space and the exit wave in real space. Hence, the basic steps for one iteration can be summarised as in **Fig. S1** and described as follows:

1. An initial estimate of the exit wave $\psi_{ex}(\boldsymbol{r})$ is generated, where $\boldsymbol{r}$ is a two-dimensional vector in real space. An exit wave $\psi_{ex}\left( \boldsymbol{r} \right)$ with unity amplitude and zero phase was used as the initial estimate. We further assumed that the input images can be represented as *m × m* matrices. In this formalism the initial exit wave $\psi_{ex}(\boldsymbol{r})$ needs to be upsampled to *n × n* pixels to provide a sufficiently large array to stitch the shifted information transfer domains (arising from the illumination tilt in Fourier space). When the Nyquist sampling criterion is satisfied, the relationship between *m* and *n* is given by: $n >m+\left| \boldsymbol{k}_{\boldsymbol{\tau}} \right|(m*p)$ where $p$ is the pixel size and $\boldsymbol{k}_{\boldsymbol{\tau}}$ is the two-dimensional wave vector of the tilted incident illumination direction. For example, if the input image size is *m × m* = 1024*×*1024 pixels, and the amplitude of the beam tilt wave vector is $\left| \boldsymbol{k}_{\boldsymbol{\tau}} \right|=5 {nm}^{-1}$, for a pixel size of 0.1 nm/pixel, the image size of the initial exit wave should be larger than 1536*×*1536 pixels. For simplicity we have used an initial estimate with two-fold upsampling (*n* $=$ *2m*). A numerical image size of 2m × 2m pixels for the exit wave $\psi_{ex}(\boldsymbol{r)}$ was chosen to ensure a sufficiently large array for stitching the shifted information transfer domains in Fourier space as shown in Supplementary **Fig. S6(a)**. The effective reconstructed region (synthetic aperture) in Fourier space is defined by these stitched domains, as illustrated in **Fig. S6(b)** and **(c)**. The tilt magnitudes and other calculation parameters used in **Fig. S6(b)** and **(c)** are the same as those used in **Fig. 3** and **Fig.5** in the main text. Based on these calculations, the effective reconstructed pixel numbers in **Fig. S6(b)** and **(c)** are approximately 1.2m^2^ and 0.7m^2^, respectively.

When the data does not meet the Nyquist sampling criterion, an upsampling scheme can be included in the reconstruction to suppress unwanted aliasing. The details of this upsampling scheme are described in **Text S3**. In summary, we assume an upsampling ratio $l$, and therefore the upsampled input image size is given as $m'$ × $m'$ where $m^{'}=m*l$. For this condition, $n >m'+\left| \boldsymbol{k}_{\boldsymbol{\tau}} \right|(m*p))$ needs to be satisfied.

1. The *j^th^* exit wave $\Psi_{{ex}_{j}}(\boldsymbol{k},\boldsymbol{k}_{\boldsymbol{\tau}})$ is obtained from the initial exit wave $\Psi_{ex}(\boldsymbol{k})$ in Fourier space. Here, $\Psi_{{ex}_{j}}(\boldsymbol{k},\boldsymbol{k}_{\boldsymbol{\tau}})$ is one Fourier domain in $\Psi_{ex}(\boldsymbol{k})$ with its position defined by the wave vector of the tilted incident beam $\boldsymbol{k}_{\boldsymbol{\tau}}$. The index *j* indicates the update corresponding to the *j^th^* tilted illumination.
2. The *j^th^* image wave is generated in real space as: $\psi_{{im}_{j}}(\boldsymbol{r,}\boldsymbol{k}_{\boldsymbol{\tau}})$ = $\mathcal{F}^{-1}(\Psi_{im_{j}}(\boldsymbol{k,}\boldsymbol{k}_{\boldsymbol{\tau}}))$ = $\mathcal{F}^{-1}[\Psi_{{ex}_{j}}\left( \boldsymbol{k,}\boldsymbol{k}_{\boldsymbol{\tau}} \right)w(\boldsymbol{k})]$ with $w(\boldsymbol{k})$ the wave transfer function.
3. The amplitude of the *j^th^* image wave is updated from the recorded *j^th^* image $I_{j}(\boldsymbol{r})$, while keeping the phase unchanged: $\psi_{{im}_{j}}^{'}\left( \boldsymbol{r,}\boldsymbol{k}_{\boldsymbol{\tau}} \right)= \sqrt{I_{j}(\boldsymbol{r})}\cdot\frac{\psi_{{im}_{j}}\left( \boldsymbol{r,}\boldsymbol{k}_{\boldsymbol{\tau}} \right)}{\left| \psi_{{im}_{j}}\left( \boldsymbol{r,}\boldsymbol{k}_{\boldsymbol{\tau}} \right) \right|}$.
4. The updated *j^th^* image wave is calculated in Fourier space as: $\Psi_{{im}_{j}}^{'}\left( \boldsymbol{k,}\boldsymbol{k}_{\boldsymbol{\tau}} \right)=\mathcal{F}(\psi_{{im}_{j}}^{'}\left( \boldsymbol{r,}\boldsymbol{k}_{\boldsymbol{\tau}} \right))$.
5. The exit wave is updated within the PIE algorithm as:

$\Psi_{{ex}_{j}}^{'}\left( \boldsymbol{k}\boldsymbol{,}\boldsymbol{k}_{\boldsymbol{\tau}} \right)=\Psi_{{ex}_{j}}\left( \boldsymbol{k}\boldsymbol{,}\boldsymbol{k}_{\boldsymbol{\tau}} \right)+ \alpha\frac{w^{*}\left( \boldsymbol{k} \right)}{\left| w\left( \boldsymbol{k} \right) \right|_{max}^{2}}(\Psi_{{im}_{j}}^{'}\left( \boldsymbol{k}\boldsymbol{,}\boldsymbol{k}_{\boldsymbol{\tau}} \right)-\Psi_{{im}_{j}}\left( \boldsymbol{k}\boldsymbol{,}\boldsymbol{k}_{\boldsymbol{\tau}} \right))$

$\text{where }\alpha$ is the updating step size and $w^{*}\left( \boldsymbol{k} \right)$ is the conjugate of $w(\boldsymbol{k})$. A step decay schedule was applied to assist convergence of the algorithm and to avoid local minima. The decay ratio used in this work was 0.5 for every 10 iterations which provide effective convergence after 50 total iterations.

1. Steps 2–6 are repeated to include all other tilted illumination images in the update of the exit wave.

Text S3. Beam tilt calibration

Calibration of the beam tilt magnitude and orientation is essential for ptychographic data acquisition and reconstruction. Tilt calibration was carried out in diffraction mode with the same illumination convergence used for data collection. The camera length was calibrated using known spacings in diffraction patterns recorded from a polycrystalline gold film. Diffraction datasets were collected with beam tilts applied in four approximately orthogonal directions corresponding to the tilt coil axes (+x, -x, +y, -y), using five tilt magnitudes per direction with a constant tilt step size. The position of the direct beam was used to measure the tilt magnitude and the orientation relationship between the beam tilt and image/detector coordinates. The measured calibrations are given in **Fig. S8** and **Table S2**.

Text S4. Upsampling

For a frequency ($f_{inf}$) that is higher than the Nyquist frequency ($f_{Nyq}$) in the recorded image, aliasing will occur. As for all imaging systems, the field of view (FOV) and resolution is an intrinsic trade off in TEM^6^. This implies that when the magnification is decreased to achieve a large FOV, the image resolution will be limited by the Nyquist sampling frequency (determined by the detector pixel pitch) and further constrained by aliasing. Hence, reconstruction of the exit wave from an eFP dataset that does not meet the Nyquist sampling criterion (**Fig. S9**), will show aliasing artifacts in the recovered exit wave as shown in **Fig. S10**, **(b1)-(b2)** and **(d1)-(d2)**. To overcome this, an upsampling scheme was applied to the update of the exit wave within the PIE algorithm. This is related to an upsampling scheme previously used in X-ray ptychography^7^ and optical Fourier ptychography^8^. The key feature of this scheme is the assumption of a large synthetic detector (*m’ × m’* pixels) with a reduced pixel size that satisfies the Nyquist criterion and the use of the original data (*m × m* pixels) to update the amplitude of the upsampled and calculated image wave. In the amplitude update, an amplitude correction matrix $C_{m',m'}=\sqrt{(U_{lanczos3} \left\{ \frac{I_{m,m}}{\left| O_{m,m} \right|_{s}^{2}} \right\})}$ was used, where the $I_{m,m}$ is the recorded image intensity, $\left| O_{m,m} \right|_{s}^{2}$ is the calculated and binned intensity from the calculated image wave, and $U_{lanczos3}$ is a lanczcos-3 interpolation. This amplitude correction matrix was applied to the calculated image wave, replacing step 4 (**Text S2**), as $\psi_{{im}_{j}}^{'}\left( \boldsymbol{r,}\boldsymbol{k}_{\boldsymbol{\tau}} \right)=\psi_{{im}_{j}}\left( \boldsymbol{r,}\boldsymbol{k}_{\boldsymbol{\tau}} \right)\cdot C_{m',m'}$. Using this upsampling scheme, aliasing artifacts are significantly reduced as shown in **Fig. S10**, **(c1)-(c2)** and **(e1)-(e2)**. However, aliasing cannot be eliminated, especially at the high spatial frequencies as highlighted in **Fig. S10**. To mitigate this, a low pass filter with a highest frequency pass of 2/3$f_{Nyq}$ can be applied to the final reconstructed exit wave as is commonly used in the processing of conventional single particle cryo-EM images to avoid interpolation errors^9^.


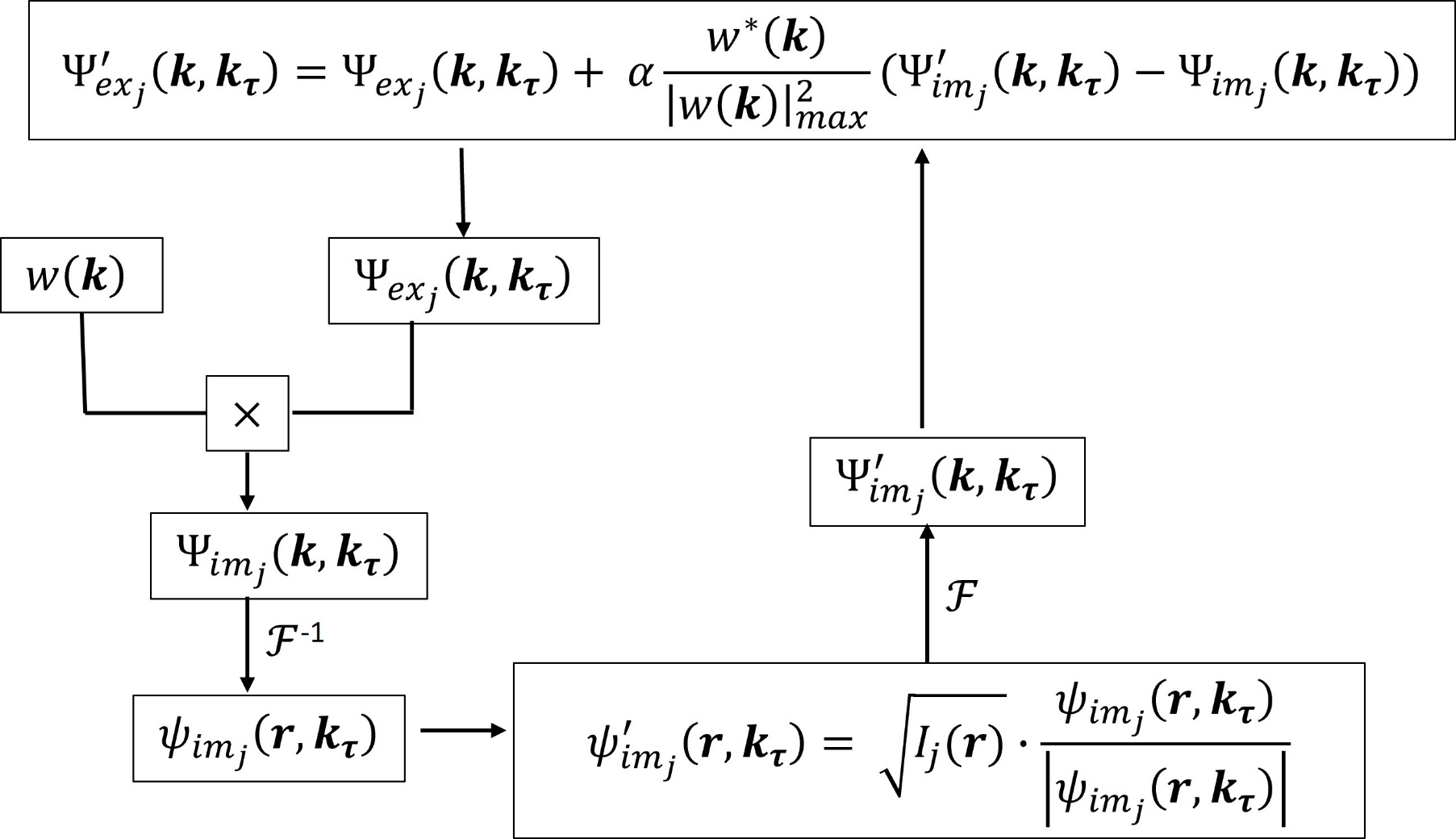


**Fig. S1. Flowchart of eFP exit wave reconstruction using the modified PIE algorithm.**


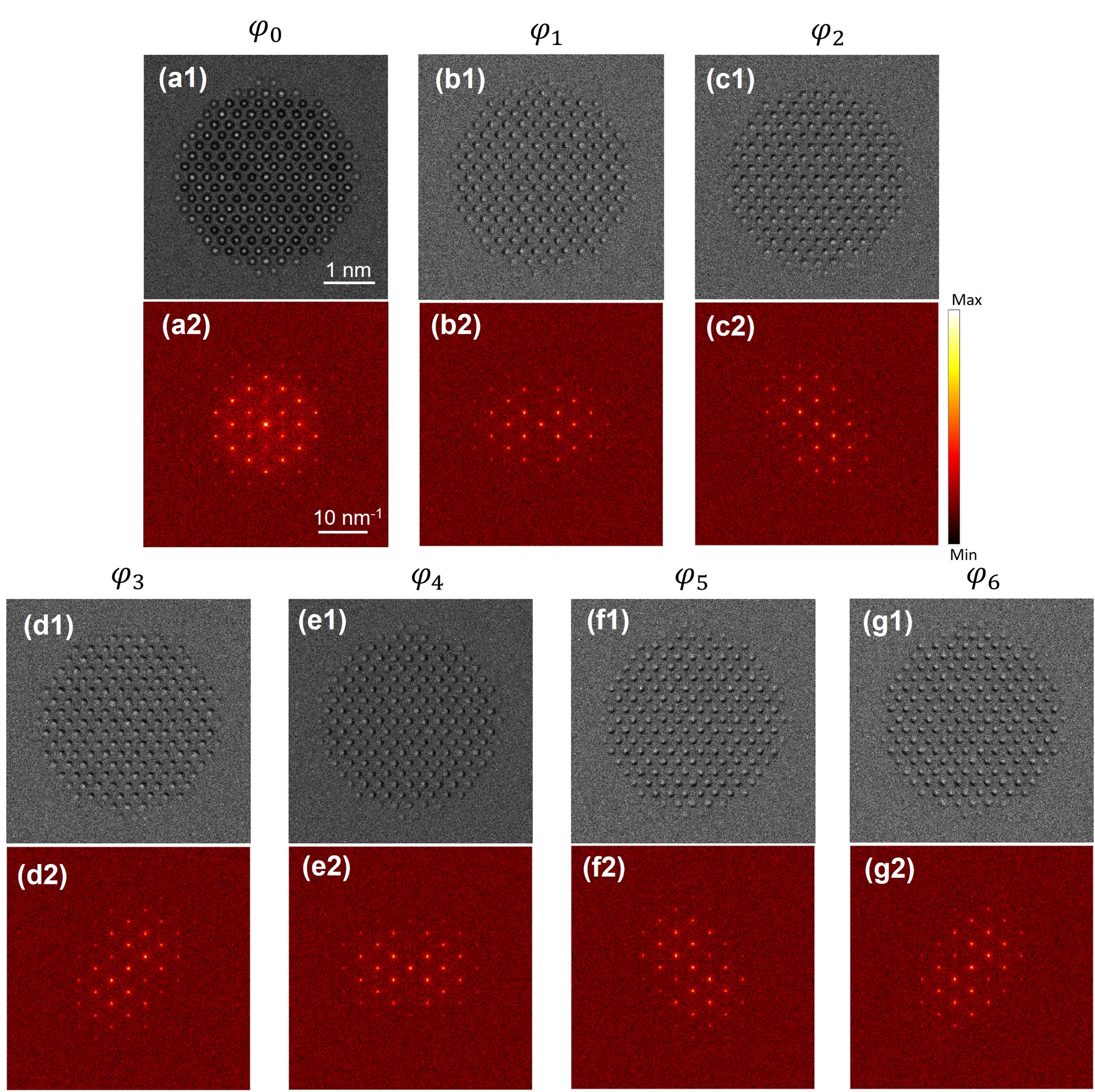


**Fig. S2. Simulated gold particle data at a beam tilt magnitude of 10.0 mrad. (a1)-(g1)** Axial and six tilted-illumination images with $\varphi_{n}$ indicating the illumination tilts; $\varphi_{0}$ corresponds to axial illumination, and $\varphi_{1}$- $\varphi_{6}$ to six tilted illuminations with evenly spaced azimuths and a constant tilt magnitude of 10.0 mrad. **(a2)-(g2)** Corresponding power spectra calculated from the amplitude of simulated image intensities. **(a1)-(g1)** are displayed with the scale bar shown in **(a1)**. Power spectra **(a2)-(g2)** are displayed with the scale bar shown in **(a2)**. The intensity of all the power spectra are weighted by a power of 0.2 for better visualisation of high-frequency information. The total fluence used was 4.6×10^5^ e^-^/nm^2^.

**
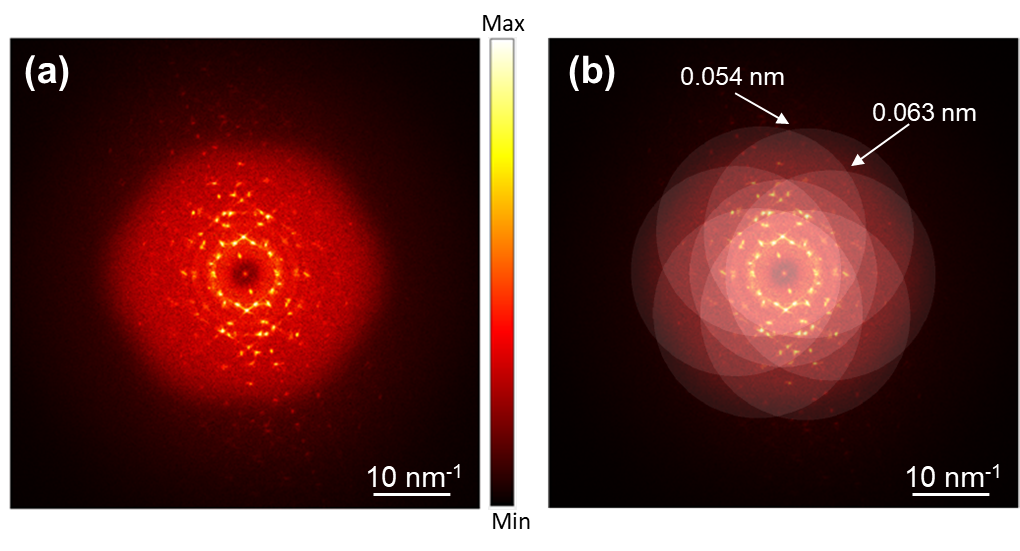
**

**Fig. S3. Power spectrum of the reconstructed phase and corresponding synthetic aperture. (a)** Power spectrum of reconstructed phase, as shown in Fig. 3 of the main text. **(b)** Spectrum with the corresponding synthetic aperture overlaid. The synthetic aperture is defined by a combination of the amplitude of axial wave transfer function and effective wave transfer function under tilted illuminations with a limit where the information transfer falls below 10%.


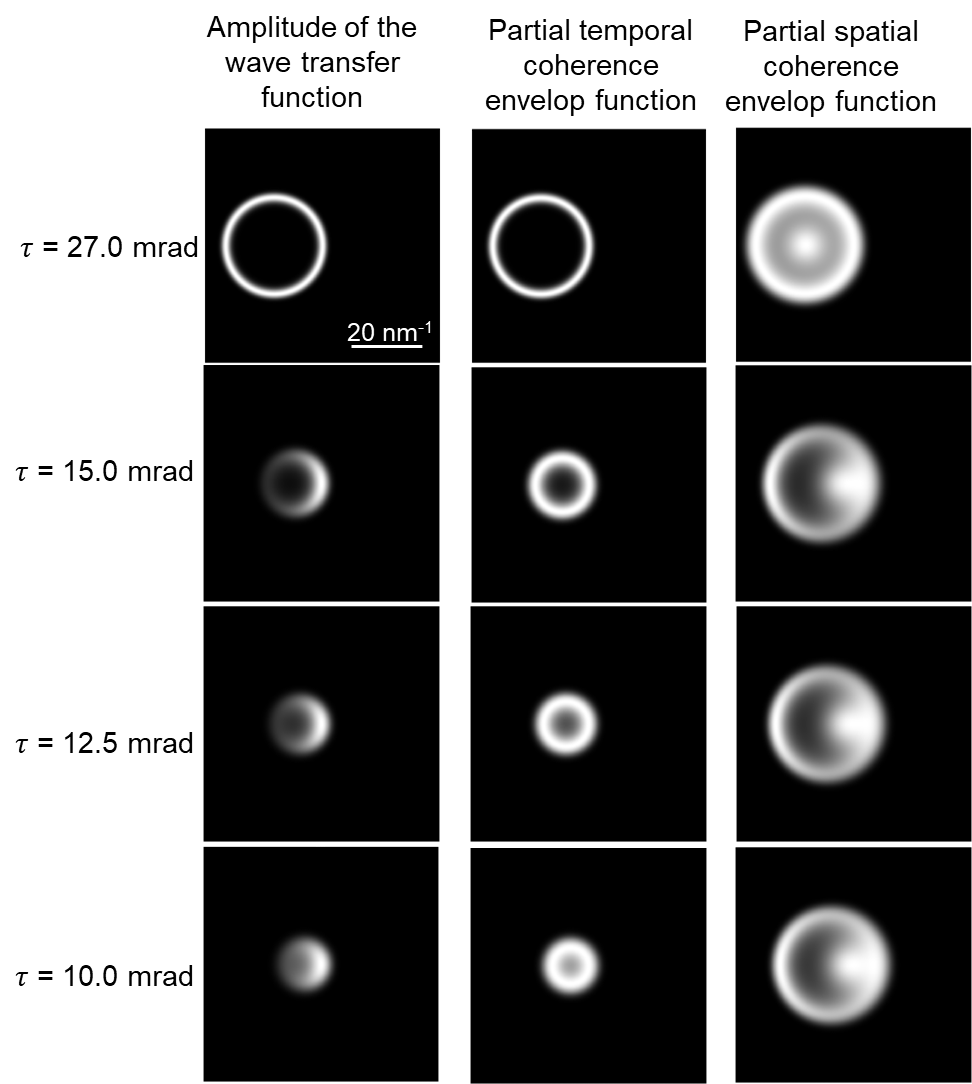

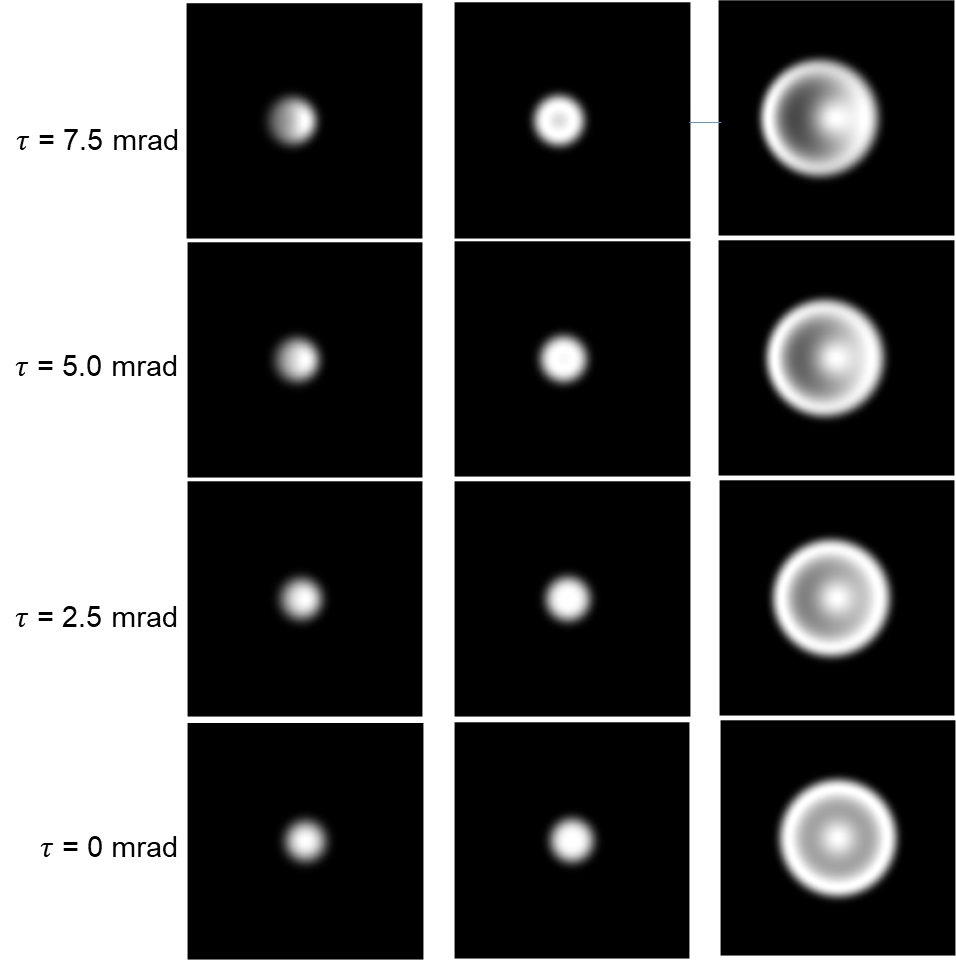


**Fig. S4. Effective wave transfer function and partial temporal and spatial coherence envelop functions calculated at different tilt magnitudes.** The calculations assume $C_{1}$ = -2000 nm and $C_{3}$ = 2.7 mm, with other parameters identical to those used in the apoferritin simulation described in the main text (**Table 1**). All images share the same scale bar shown in the top right image.


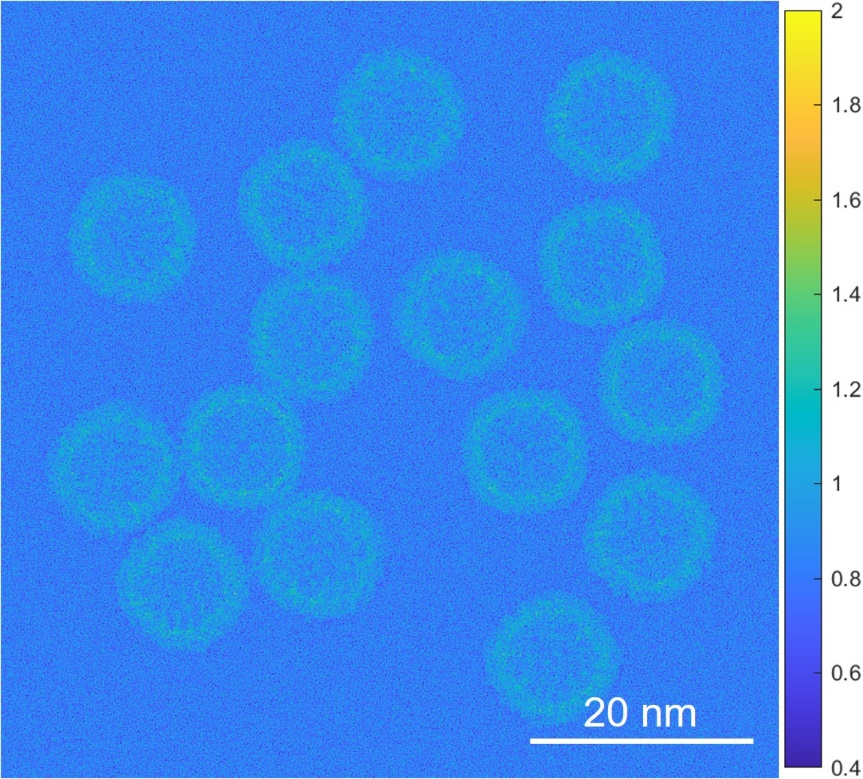


**Fig. S5. Simulated phase of apoferritin particles used as the ground truth for evaluating the quality of the reconstructed phase.** Phase scale in radians.


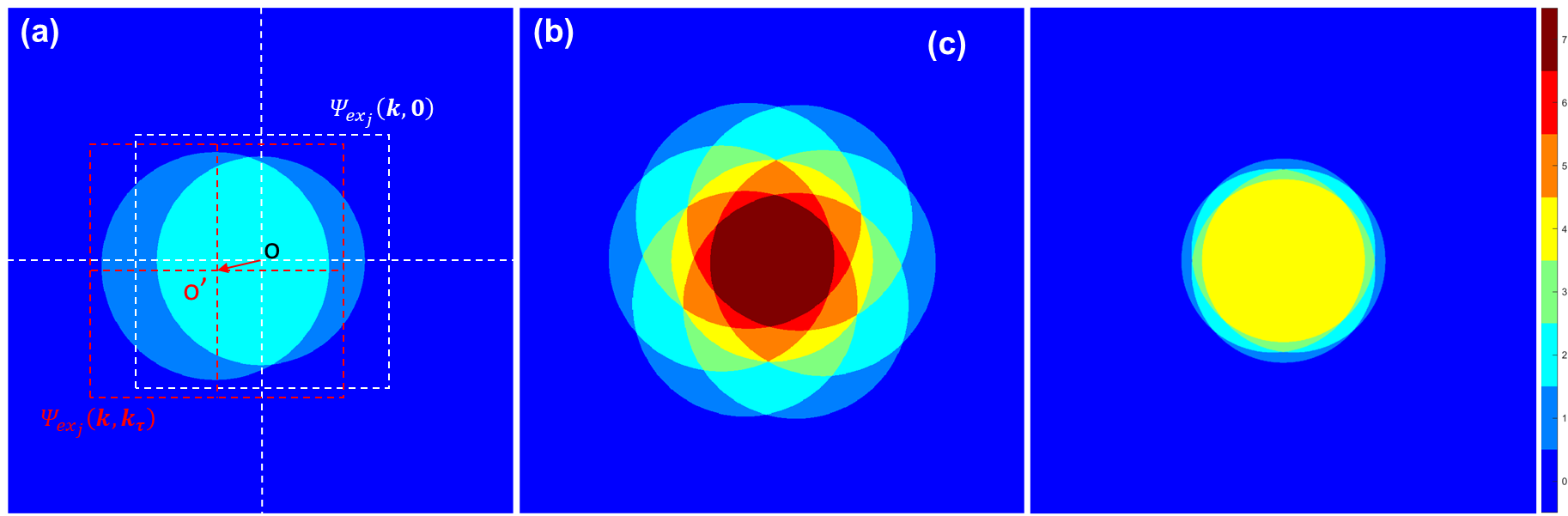


**Fig. S6. Schematic illustration of the synthetic aperture and measurement redundancy**. **(a)** Information corresponding to axial illumination (white dashed-square, $\Psi_{{ex}_{j}}\left( \boldsymbol{k,0} \right)$) and that corresponding to a single tilted illumination image (red dashed-square, $\Psi_{{ex}_{j}}\left( \boldsymbol{k,}\boldsymbol{k}_{\boldsymbol{\tau}} \right)$). **(b)** and **(c)** Synthetic apertures used in the reconstructions in **Fig. 3** and **Fig. 5** of the main text. Colour bar indicates measurement redundancy across different spatial frequency regions.


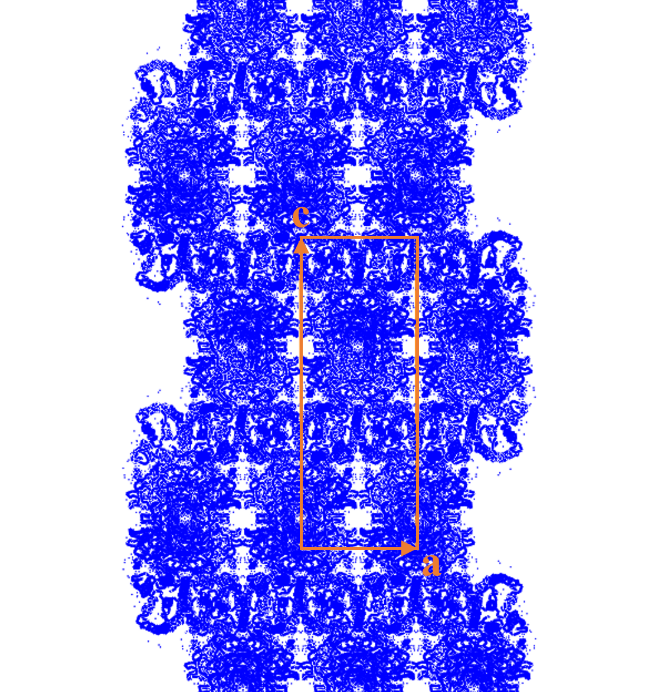


**Fig. S7. Cry11Aa crystal structure projected along [010].**

**
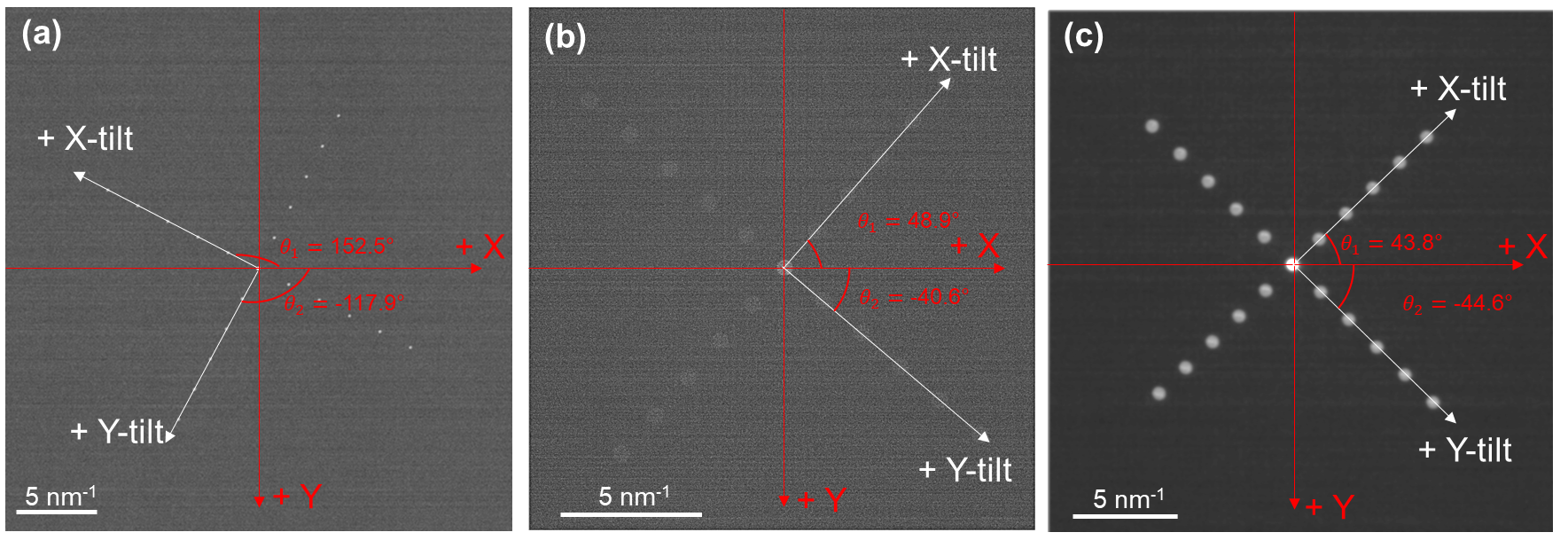
**

**Fig. S8. Tilt calibration for the experimental datasets. (a)** For the gold particle dataset collected on a JEM-ARM300F2. **(b)** For the rotavirus dataset collected on a JEM-Z300FSC. **(c)** For the Cry11Aa dataset collected on JEM-Z300FSC. In all cases the direct beam was defocused to protect the detector.


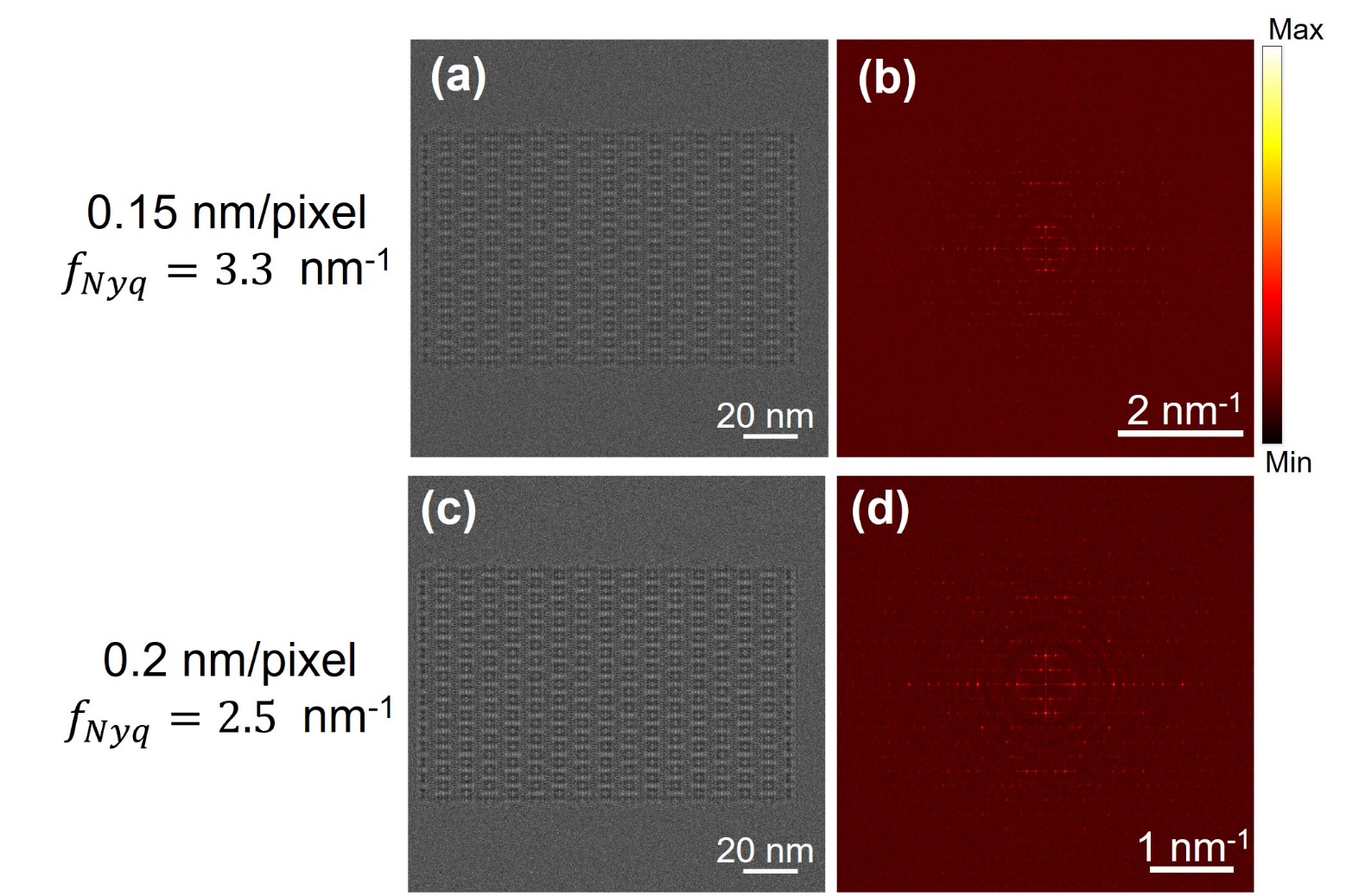


**Fig. S9. Simulated Crys11Aa data with different pixel samplings. (a)** and **(c)** tilted-illumination images (tilt magnitude 1.9 mrad) with a pixel sampling of 0.15 nm/pixel and 0.2 nm/pixel, respectively. **(b)** and **(d)** power spectra calculated from **(a)** and **(c)**. The total electron fluence is 3×10^3^ e^-^/nm^2^. Data simulation details are as used in **Fig. 5** in the Main text, but with different pixel sampling and electron fluence. These represent two examples where the input eFP data fails to meet the Nyquist sampling criterion.


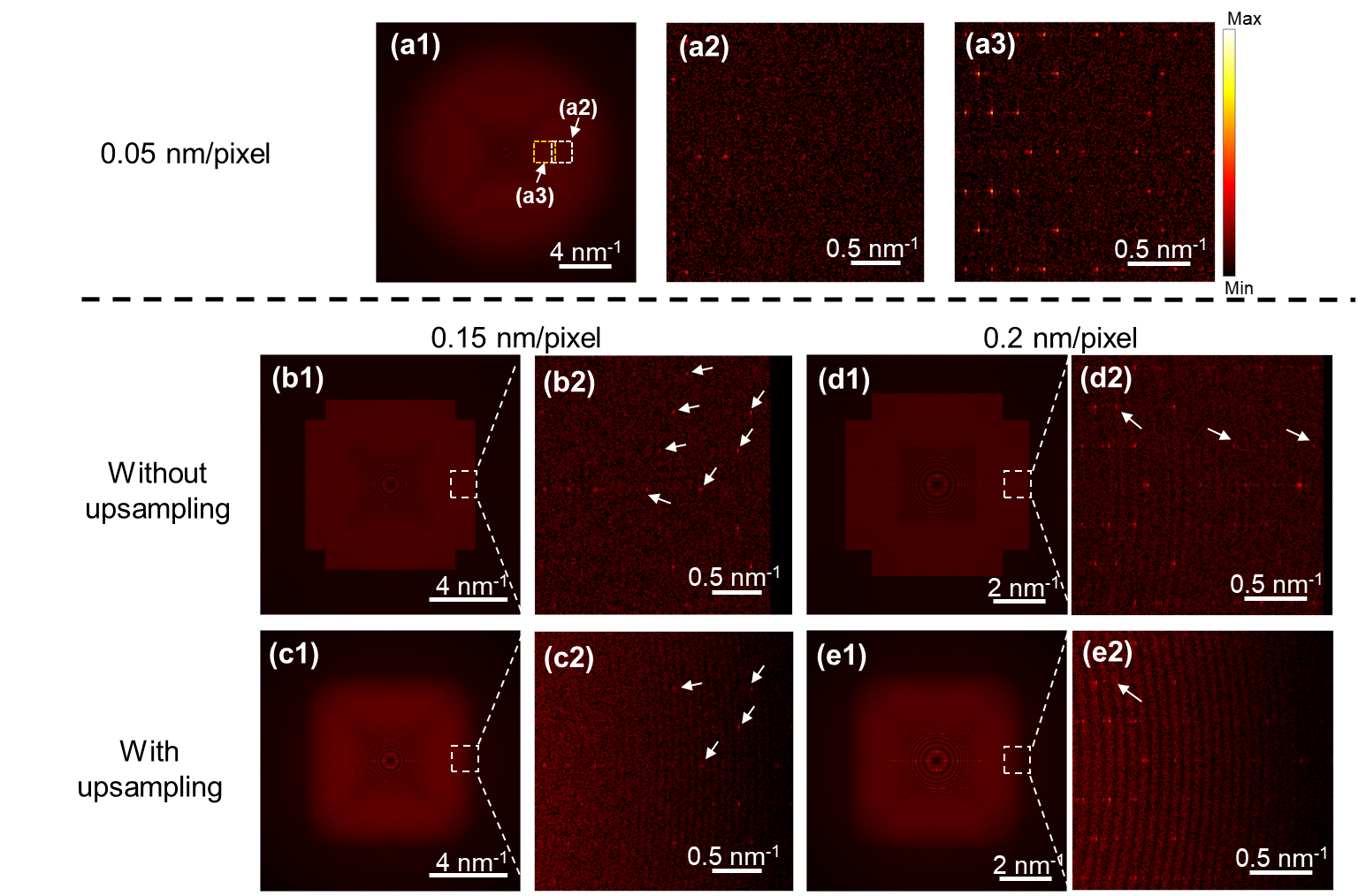


**Fig. S10. Upsampling for anti-aliasing. (a1)** Power spectrum calculated from the recovered phase of the simulated Cry11Aa dataset with a pixel sampling of 0.05 nm/pixel. **(a2)** and **(a3)** Enlarged views of the marked regions in (**a1**). **(b1)** Power spectrum calculated from the recovered phase with a pixel sampling of 0.15 nm/pixel for reconstruction without upsampling. **(b2)** Enlarged region marked in **(b1)**. **(c1)** and **(c2)** as for **(b1)** and **(b2)**, but with upsampling. **(d1)** Power spectrum calculated from the recovered phase from the dataset with a pixel sampling of 0.2 nm/pixel without upsampling. **(d2)** Enlarged region marked in **(d1)**. **(e1)** and **(e2)** as for **(d1)** and **(d2)**, but with upsampling. **(a1)**-**(a3)** are used as a comparison with **(b1)**-**(e2)**. **(a2)** and **(a3**) correspond to the same frequency regions as those shown in **(b2)**-**(c2)** and **(d2)**-**(e2)**, respectively. The arrow marks artefacts due to aliasing.

Table S1. Corrected axial aberration coefficients to third order in the wave aberration function

| Aberrations | Amplitude | Azimuth (degree) |
| --- | --- | --- |
| Defocus, *C*_1_ (nm) | 5 |  |
| Two-fold astigmatism, *A*_1_ (nm) | 2.0 | -89.8 |
| Three-fold astigmatism, *A2 (nm)* | 30.1 | -11.0 |
| Axial coma*, B*_2_ (nm) | 22.4 | -41.9 |
| Four-fold astigmatism, *A3* (nm) | 162 | -19.5 |
| Axial star aberration, *S3 (nm)* | 1256 | -27.5 |
| Spherical aberration, *C*_3_ (mm) | 0.0015 |  |

Table S2. Beam tilt calibration

| Dataset | Tilt unit (mrad/143 bytes) | $\theta_{1}$***** (degree) | $\theta_{2}$***** (degree) |
| --- | --- | --- | --- |
| Gold particle | 0.9 | 152.5 | -117.9 |
| Rotavirus | 3.8 | 48.9 | -40.6 |
| Cry11Aa crystal | 3.8 | 43.8 | -44.6 |

*$\boldsymbol{\theta}_{\boldsymbol{1}}$ is defined as the angle between the image x-axis and the beam + x-tilt; $\boldsymbol{\theta}_{\boldsymbol{2}}$ is defined as the angle between the image x-axis and the beam + y-tilt. This definition is illustrated in Fig. S5.

References

1. Kirkland, A. I. & Meyer, R. R. “Indirect” High-Resolution Transmission Electron Microscopy: Aberration Measurement and Wavefunction Reconstruction. *Microsc. Microanal.* **10**, 401–413 (2004).

2. Haigh, S. J., Sawada, H. & Kirkland, A. I. Optimal tilt magnitude determination for aberration-corrected super resolution exit wave function reconstruction. *Phil. Trans. R. Soc. A.* **367**, 3755–3771 (2009).

3. Saxton, W. O. Observation of lens aberrations for very high‐resolution electron microscopy. I. Theory. *J. Microsc.* **179**, 201–213 (1995).

4. Rodenburg, J. M. & Faulkner, H. M. L. A phase retrieval algorithm for shifting illumination. *Appl. Phys. Lett.* **85**, 4795–4797 (2004).

5. Hüe, F., Rodenburg, J. M., Maiden, A. M., Sweeney, F. & Midgley, P. A. Wave-front phase retrieval in transmission electron microscopy via ptychography. *Phys. Rev. B.* **82**, 121415 (2010).

6. Zheng, G., Shen, C., Jiang, S., Song, P. & Yang, C. Concept, implementations and applications of Fourier ptychography. *Nat. Rev. Phys.* **3**, 207–223 (2021).

7. Batey, D. J. *et al.* Reciprocal-space up-sampling from real-space oversampling in x-ray ptychography. *Phys. Rev. A.* **89**, 043812 (2014).

8. Sun, J., Chen, Q., Zhang, Y. & Zuo, C. Sampling criteria for Fourier ptychographic microscopy in object space and frequency space. *Opt. Express.* **24**, 15765 (2016).

9. Cheng, Y., Grigorieff, N., Penczek, P. A. & Walz, T. A Primer to Single-Particle Cryo-Electron Microscopy. *Cell.* **161**, 438–449 (2015).
